# Acetic acid increases the phage-encoded enterotoxin A expression in *Staphylococcus aureus*

**DOI:** 10.1186/1471-2180-10-147

**Published:** 2010-05-20

**Authors:** Nina Wallin-Carlquist, Rong Cao, Dóra Márta, Ayla Sant'Ana da Silva, Jenny Schelin, Peter Rådström

**Affiliations:** 1 Applied Microbiology, Lund Institute of Technology, Lund University, Lund, Sweden; 2 Dept. of Microbiology and Biotechnology, Faculty of Food Science, Corvinus University of Budapest, Budapest, Hungary; 3 Dept. of Biochemistry, Chemistry Institute, Federal University of Rio de Janeiro, Rio de Janeiro, Brazil

## Abstract

**Background:**

The effects of acetic acid, a common food preservative, on the bacteriophage-encoded enterotoxin A (SEA) expression and production in *Staphylococcus aureus *was investigated in pH-controlled batch cultures carried out at pH 7.0, 6.5, 6.0, 5.5, 5.0, and 4.5. Also, genomic analysis of *S. aureus *strains carrying *sea *was performed to map differences within the gene and in the temperate phage carrying *sea*.

**Results:**

The *sea *expression profile was similar from pH 7.0 to 5.5, with the relative expression peaking in the transition between exponential and stationary growth phase and falling during stationary phase. The levels of *sea *mRNA were below the detection limit at pH 5.0 and 4.5, confirmed by very low SEA levels at these pH values. The level of relative *sea *expression at pH 6.0 and 5.5 were nine and four times higher, respectively, in the transitional phase than in the exponential growth phase, compared to pH 7.0 and pH 6.5, where only a slight increase in relative expression in the transitional phase was observed. Furthermore, the increase in *sea *expression levels at pH 6.0 and 5.5 were observed to be linked to increased intracellular *sea *gene copy numbers and extracellular *sea-*containing phage copy numbers. The extracellular SEA levels increased over time, with highest levels produced at pH 6.0 in the four growth phases investigated. Using mitomycin C, it was verified that SEA was at least partially produced as a consequence of prophage induction of the *sea*-phage in the three *S. aureus *strains tested. Finally, genetic analysis of six *S. aureus *strains carrying the *sea *gene showed specific *sea *phage-groups and two versions of the *sea *gene that may explain the different *sea *expression and production levels observed in this study.

**Conclusions:**

Our findings suggest that the increased *sea *expression in *S. aureus *caused by acetic acid induced the *sea*-encoding prophage, linking SEA production to the lifecycle of the phage.

## Background

Staphylococcal enterotoxins (SEs) are extracellular proteins, produced mainly by *Staphylococcus aureus*, causing food intoxication when ingested. Staphylococcal food poisoning (SFP) was the fourth most common causative agent in food-borne illness within the EU in 2008 [1]. It is associated with food, generally rich in protein, which requires extensive manual handling, often in combination with inadequate heating and/or inappropriate storage of the food [2,3]. To date, 21 staphylococcal enterotoxins or enterotoxin-like proteins (SEA-SEE, SEG-SEV), excluding variants, have been identified. These SE genes are widely disseminated by several mobile genetic elements leading to variations in the SE expression behavior among enterotoxigenic staphylococci [2-5]. The expression of a number of the enterotoxins including SEB, SEC, and SED is to some extent known to involve regulatory systems such as the accessory gene regulator (Agr), the staphylococcal accessory regulator (Sar) and the repressor of toxin (Rot) [6]. However, we still have limited information about SEA, the toxin considered to be mainly responsible for staphylococcal food poisoning outbreaks [7-11]. The SEA gene is carried in the bacterial genome by a polymorphic family of temperate bacteriophages [12-14]. Recent studies of *S. aureus *strain MSSA476 have shown that mitomycin C (MC) induction of ΦSa3ms, resulted in increased transcription of enterotoxins SEA, SEG, and SEK and the fibrinolytic enzyme staphylokinase (Sak) carried by the prophage [14]. Although, it is still unclear if the increased transcription of these virulence determinants lead to increased amounts of SE proteins. Furthermore, identification of the environmental parameters that control the expression of SEA in food, and the mechanism by which these signals are transduced to bring about changes in gene expression, are very limited. This knowledge is crucial for understanding the potential of *S. aureus *to cause food poisoning.

Acetic acid is a weak organic acid often used in the food industry as a preservative due to its antagonistic effect on bacterial pathogens [15]. Weak acids have the ability to pass through the cell membrane in the undissociated form. Once inside the cell, the acid dissociates in the more alkaline interior, lowering the intracellular pH of the cell. A decrease in intracellular pH can lead to the damage of macromolecules (e.g. proteins and DNA) and the cell membrane, and have a negative effect on cell maintenance [16,17]. Also, the anion of the acid is accumulated intracellularly, increasing turgor pressure [18]. Acetic acid has been found to be more inhibitory to the growth of *S. aureus *than lactic acid, citric acid, phosphoric acid and hydrochloric acid, respectively [19]. Also, acetic acid has been found to almost completely inhibit SEA formation in brain heart infusion (BHI) broth when added gradually over time [20].

In the present study, the effects of acetic acid on *S. aureus *growth, *sea *expression and SEA production were investigated in four growth phases. Furthermore, the relationship between SEA production and the lifecycle of the phage carrying the toxin gene was determined. Finally, genomic analysis of *S. aureus *strains carrying *sea *was performed to map differences within the gene and in the temperate phage carrying *sea*.

## Results

### Effects of acetic acid on *sea *expression and SEA production in *S. aureus *Mu50

**Figure 1:**
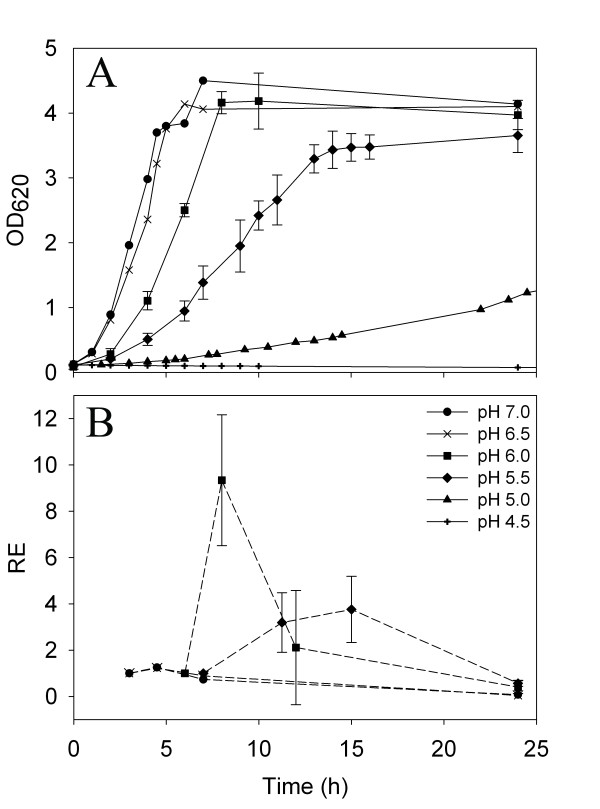
**Growth and relative *sea *levels of *S. aureus *Mu50 when grown at different pH levels**. (A) Growth curves determined by OD measurements at 620 nm at pH 7.0, 6.5, 6.0, 5.5, 5.0, and 4.5. (B) Relative expression (RE) of *sea *at pH 7.0, 6.5, 6.0, and 5.5. Solid and dashed lines represent growth and RE, respectively. For pH 6.0 and 5.5, the mean and standard deviations of independent batch cultures; two and three, respectively, is displayed.

Batch cultures of *S. aureus *Mu50, harboring the *sea*-containing Φ42-like prophage ΦMu50A [21], were carried out at controlled pH levels of 7.0, 6.5, 6.0, 5.5, 5.0, and 4.5 (Figure 1A). Acetic acid was used to set the pH to investigate the effects of acetic acid on growth, relative *sea *expression and extracellular SEA levels during all stages of growth. The maximal growth rate of *S. aureus *Mu50 was highest at pH 7.0 and decreased with decreasing pH (Figure 1A). Batch cultivations performed at lower pH values showed that pH 5.0 was highly growth-inhibitory, with only a modest increase in optical density, OD, and viable cells in the late stationary growth phase, and that pH 4.5 was too toxic; < 1% of the starting inoculum was viable after 24 h. The relative *sea *expression pattern was similar at all tested pH levels that allowed expression analysis (Figure 1B); the highest relative levels of *sea *mRNA were found in the transitional phase and fell during the stationary growth phase. Small increases in *sea *expression were found in the transitional phase at pH 7.0 and 6.5. However, relative *sea *expression in the transitional phase at pH 6.0 (n = 2) and 5.5 (n = 3) were high, nine and four times higher, respectively, than in the exponential growth phase. At pH 5.5, extended *sea *mRNA expression was observed with the peak associated with the transitional phase. However, *sea *mRNA was not possible to detect at pH 5.0 or 4.5.

**Figure 2:**
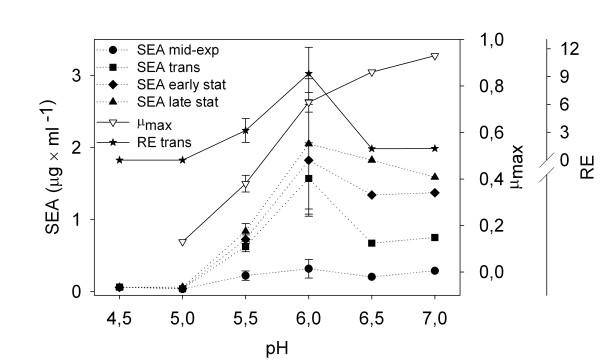
**SEA levels, growth rate and *sea *expression of *S. aureus *Mu50 at different pH levels**. Extracellular SEA levels in the mid-exponential, the transitional, the early stationary, and late stationary growth phase; maximal growth rate (μ_max_), and relative *sea *levels (RE) in the transitional phase. At pH 4.5 the SEA values are after 10, 24 and 30 h of growth, shown in the figure as transitional, early stationary and late stationary phase samples, respectively. The values at pH 6.0 and 5.5 are the average and standard deviations of two and three independent batch cultures, respectively.

Extracellular SEA was detected in all cultivations of *S. aureus *Mu50 and the levels increased over time at tested pH levels allowing growth (Figure 2). The SEA levels increased from pH 7.0 to 6.0 and decreased significantly at lower pH levels, i.e. pH 5.5, 5.0 and 4.5. The specific extracellular SEA concentrations (i.e. the extracellular SEA concentrations divided by the value of the OD at that point in time) correlating the SEA production to growth, showed the same trend. The specific SEA concentrations were 100, 450, 510, 210, 40, and 870 ng per ml and OD unit for pH 7.0, 6.5, 6.0, 5.5, 5.0, and 4.5, respectively. The specific SEA concentration at pH 4.5 is misleading since the culture was not growing.

### Phage-associated *sea *expression

**Figure 3:**
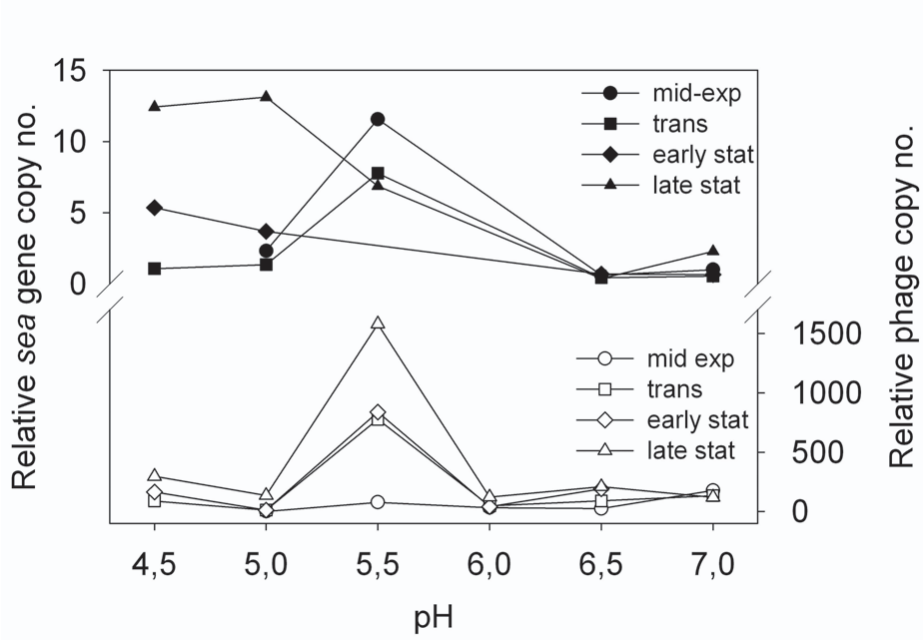
**Change in *sea *gene copy number and *sea*-carrying phage copy number of *S. aureus *Mu50**. The relative *sea *gene copy numbers and phage copy numbers in the mid-exponential, the transitional, the early stationary, and the late stationary growth phase of *S. aureus *Mu50 at different pH levels; black symbols are the relative *sea *gene copy numbers and white symbols are the relative phage copy numbers. At pH 4.5, the SEA values are after 10, 24 and 30 h of growth, shown in the figure as transitional, early stationary and late stationary phase samples, respectively. For pH 6.0 and 5.5, representative values of several independent batch cultures are shown.

Samples of bacterial cells and culture supernatants from *S. aureus *Mu50 were collected to determine the trends of the relative *sea *gene copy number (and thus the replicative form of the *sea-*carrying phage) and relative phage copy number in the four growth phases at different pH values (Figure 3). The relative *sea *gene copy number was low throughout the cultivations at pH 7.0 and 6.5. The *sea *gene copy number peaked at pH 5.5, being twelve times higher than at pH 7.0 in the mid-exponential growth phase, and a trend of the *sea *gene copy number decreasing over time was observed at this pH. The *sea *gene copy number increased over time at pH 5.0 and 4.5, showing markedly higher levels at the last sampling point. The phage copy number increased over time at all pH levels, with a peak at pH 5.5. In the late stationary growth phase, the phage copy number was 13 times higher at pH 5.5 than at pH 7.0.

**Figure 4:**
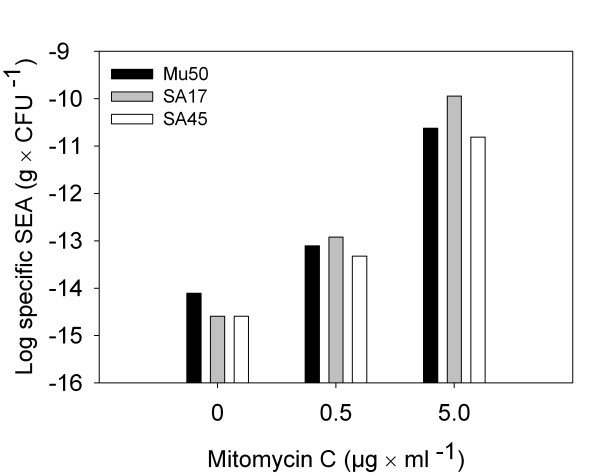
**Specific extracellular SEA levels of *S. aureus *Mu50, SA17, and SA45 after mitomycin C treatment**.

To investigate if the extracellular SEA levels were affected by prophage induction, 0.5 μg/ml or 5.0 μg/ml MC was added to exponentially growing *S. aureus *strains Mu50, SA17, and SA45 (Figure 4). The number of viable cells of strain Mu50 after three hours of growth following MC addition was reduced by two log units in cultures containing 0.5 μg/ml MC and five log units in cultures containing 5.0 μg/ml MC, compared with control cultures containing no MC. For both strains SA17 and SA45 the viable cell counts were reduced by one and four log units in cultures containing 0.5 μg/ml and 5.0 μg/ml MC, respectively (data not shown). The specific extracellular SEA levels, i.e. the extracellular SEA concentration per colony-forming unit, CFU, of *S. aureus *strains Mu50, SA17, and SA45, increased with MC concentration compared to the control cultures, being ten, 50, and 20 times higher at 0.5 μg/ml MC, and 3000, 40 000, and 6000 times higher at 5.0 μg/ml MC for Mu50, SA17, and SA45, respectively. Viable phage particles, defined as plaque forming units, were observed for strains SA17 and SA45 after MC treatment but not for Mu50 using *S. aureus *RN450 as recipient strain (for Mu50, *S. aureus *RN4220 was also tested) (data not shown).

### Effects of acetic acid on *sea *expression and SEA production in *S. aureus *SA45

**Figure 5:**
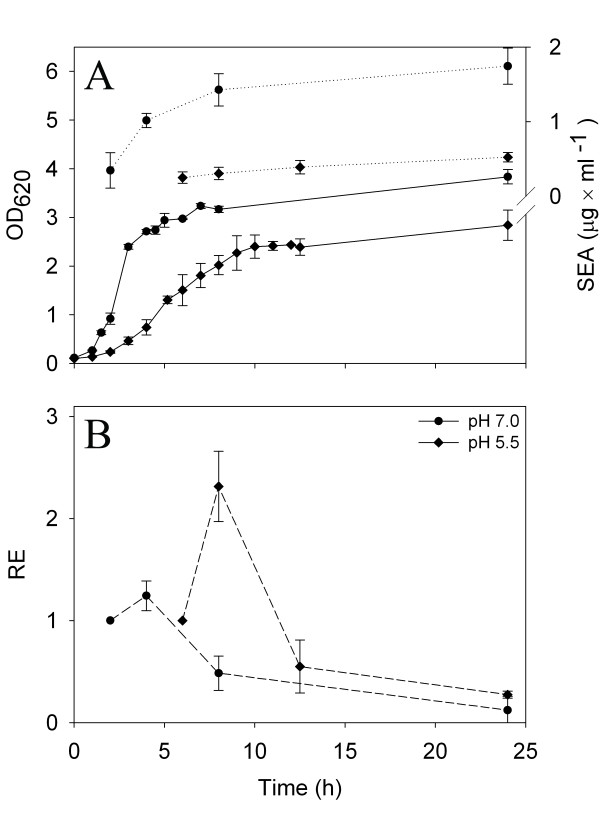
**Growth, SEA levels, and *sea *mRNA levels of *S. aureus *SA45 grown at two pH levels**. (A) Growth curves determined by OD measurements at 620 nm and extracellular SEA levels at pH 7.0 and pH 5.5. (B) Relative expression (RE) of *sea *at pH 7.0 and pH 5.5. Solid, dotted and dashed lines represents growth, SEA levels and RE, respectively. Values are the mean and standard deviations of two independent batch cultures.

To determine if the response to acetic acid was specific to strain Mu50 or a more general *S. aureus *response, a strain isolated from ham involved in a food poisoning outbreak, *S. aureus *SA45, was used to replicate the batch cultivations at pH 7.0 and pH 5.5 (Figure 5 A and B). *S. aureus *SA45 had higher maximal growth rate than *S. aureus *Mu50, but the cultures never reached the same maximum OD as Mu50. The relative *sea *expression pattern of *S. aureus *SA45 was the same as for *S. aureus *Mu50, with the highest relative *sea *levels found in the transitional phase. The *sea *mRNA levels and extracellular SEA amounts were very similar for both strains at pH 7.0. However, at pH 5.5 the relative *sea *expression in the transitional phase was 38% higher in *S. aureus *Mu50 compared to in *S. aureus *SA45 and the final extracellular SEA concentration in the *S. aureus *Mu50 cultures was 61% higher than in *S. aureus *SA45 cultures on average.

### Genetic diversity of *sea*

Nucleotide sequence analysis of *sea *and prophage regions immediately upstream and downstream of the gene was performed on the whole-genome sequenced *S. aureus *strains MRSA252 [22], MSSA476 [22], Mu3, Mu50 [21], MW2 [23], and Newman [24] to determine genetic differences that may explain the different *sea *expression and SEA production profiles observed at pH 5.5 with *S. aureus *Mu50 and SA45. Sequence alignment of the coding region of *sea *revealed two main groups of *sea*-carrying phages. Within a group the *sea *sequences showed 100% sequence similarity and between the two groups the sequence similarity was 98%. Prophages ΦMu3, ΦMu50A, ΦSa3ms, and ΦSa3mw clustered together in a *sea*-group designated *sea*_1_, while Φ252B and ΦNM3 formed a *sea *group, designated *sea*_2_.

**Figure 6:**
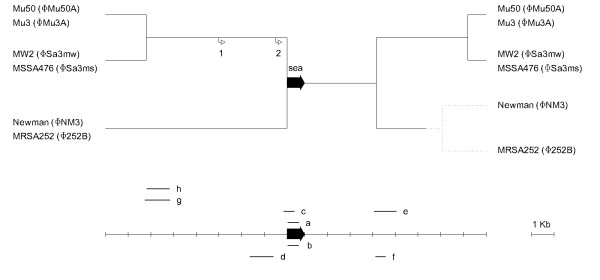
**Gene map of the *sea *virulence region of *S. aureus***. Gene map of the *sea *gene and regions immediately upstream and downstream of the gene in six different *S. aureus *strains. The map is based on nucleotide sequence analysis of the strains. Solid lines are sequences within the *sea*-carrying prophage. Dotted lines represent sequences outside the prophage region. The letters a-h indicates were PCR amplicons are located within the region; numbers 1-2 indicate transcription start sites [14].

All six phages shared a homologous region of 3.2 kb downstream of the *sea *gene containing the *sak *gene. Thereafter, the nucleotide sequences diverged, forming three subgroups of *sea *phages. The same grouping of phages was observed immediately upstream of the translational start site of *sea *(Figure 6). An analogous phage grouping was recently reported when comparing the integrase (*int*) nucleotide sequences of these bacteriophages [25]. To improve the resolution of phylogenetic analysis of these bacteriophages based on *int *genes, we repeated the *int *gene grouping (data not shown). The ΦMu3A/ΦMu50A branch was found to be closer to the Φ252B/ΦNM3 branch than to the ΦSa3ms/ΦSa3mw branch. This is in direct contrast to what was found for the *sea *gene.

In order to identify the phage- and *sea*-group of SA45, eight different regions were targeted by PCR (see Table 1 and Figure 6). This analysis showed that SA45 carries the *sea*_1_-version of the *sea *gene and belongs to the same subgroup as ΦSa3mw.

**Table 1:** Primer used for conventional PCR and results from PCR analysis of four *S. aureus *strains.

**Primer name**^**1**^	Nucleotide sequence (5' → 3')	**Primer location**^**2**^	Annealing temperature (°C)	PCR results			
				Mu50	MW2	Newman	SA45
a _forward_	TAT TCA TTG CCC TAA CGT T	789421	49	+	+	-	+
a _reverse_	CCG TCT AGC CAT AAA TTG ATC	789842					
b _forward_	TAT TCA TTG CCC TAA CGT G	783956	51	-	-	+	-
b _reverse_	CCG TCT AGC CAT AAA TTG ATT	784377					
c _forward_	GGC AAG ATG GTT ATC ATG	789043	47	*+*	*+*	-	*+*
c _reverse_	CGA TTA TTA TCA TGT AAC G	789799					
d _forward_	GTT CTG ATG AGA ACT ATG	781925	48	-	-	*+*	-
d _reverse_	CGT CTC CGC AAT TTT C	782948					
e _forward_	GGC TAT AGA TGG ATT AC	793236	47	*+*	*+*	-	*+*
e _reverse_	AGA GCT TCG TCA ATT TCA	794180					
f _forward_	GGT AGA CAA GGC AGG TAA TAG	787832	55	-	-	*+*	-
f _reverse_	GTG GAC TTC CTA CAA CGC	788235					
g _forward_	CAT TGA ATG GTT AGT TGT AC	761697	50	-	*+*	-	*+*
g _reverse_	GTC CAA GTT ATA CAT TAT CGG	762676					
h _forward_	GAA CGC GTC TAT AGA AAA G	782755	51	*+*	-	-	-
h _reverse_	GTC CAA GTT ATA CAT TAT CGG	783832					

(+) amplification occurred in PCR using the primer pair and genomic DNA from the *S. aureus *strain listed. (-) no PCR amplification was observed.

^1^Primer names indicate the physical position of PCR amplicon in Figure 6.

^2^Primer location indicates the position of the first 5'-nucleotide within the annotated genomes.

## Discussion

The genetic diversity analysis of the prophage region encoding SEA showed two main groups of genes, *sea*_*1 *_and *sea*_*2*_. To our knowledge this has not been observed before. Furthermore, Figure 6 shows that the *sea*_*1 *_and *sea*_*2 *_genes are associated with specific bacteriophages which could be further grouped based on sequence similarities within regions upstream and downstream of the *sea *gene. Borst and Betley divided enterotoxin-A-producing *S. aureus *into high-SEA producing and low-SEA producing strains [13]. The variation in SEA production was associated with differences in the prophage region immediately upstream of *sea*. The six strains analyzed here could be divided in three groups based on sequence differences in the *sea*-virulence region. However, a different grouping than for the *sea *gene was observed upon comparing the *int *gene of these phages. The *int *gene, being part of the core genome, is essential for the phage's lifecycle unlike the *sea *gene, and is therefore reflecting the evolutionary relationship among these phages. Nucleotide sequence analysis of *S. aureus *Mu50 and SA45 showed that they belong to different groups based on variations in the nucleotide sequences within the *sea*-virulence region. This division may explain the differences observed between the two strains regarding *sea *expression and SEA levels at pH 5.5.

The *sea *expression was highest in the transition from the exponential to the stationary growth phase in both *S. aureus *Mu50 and SA45 at all pH levels that allowed expression analysis, as established previously [26,27]. A boost in *sea *expression was observed in the transitional phase in *S. aureus *Mu50 at pH 6.0 and pH 5.5, which was also found in strain SA45. The same expression pattern has been found for the prophage-encoded Panton-Valentine leukocidin (PVL, *luk-PV*) of *S. aureus *[28]. Maximal expression of *luk-PV *in the late exponential growth phase was followed by a rapid decline post-exponentially. Our observation could partially be explained by the induction of the prophage carrying the toxin gene. The *sea*-phage copy numbers of *S. aureus *Mu50 at pH 6.0 remained constant during the first part of cultivation. In the late stationary growth phase, however, the number had increased four times (average increase of two biological replicates) compared to levels in early stationary growth phase. The phage copy numbers might have increased further if growth was allowed to continue. An acetic-acid induced intracellular drop in pH, leading to oxidative stress [29] would activate the SOS response system inducing the prophage [30]. Sumby and Waldor showed that upon prophage induction in *S. aureus*, the phage DNA was replicated, resulting in an increase in *sea *gene copy number, and that a second prophage-regulated *sea *promoter was also activated, resulting in increased *sea *expression [14]. Similar enhanced transcription of phage-encoded virulence genes upon prophage induction has also been observed for PVL in *S. aureus *and the Shiga toxins in *E. coli *[28,31].

Mitomycin C, a well-known prophage inducer, was used in this study. The more MC added, the more SEA was produced per CFU for all three strains tested, supporting the association between prophage induction and SEA production. However, the expected boost in extracellular SEA levels accompanying the increased *sea *mRNA levels and *sea *gene copy levels observed at pH 5.5 was not found. This could be because of the pronounced phage production at pH 5.5 seen as a rapid increase in extracellular *sea*-phage copy number (Figure 3). The window for phage-encoded SEA-biosynthesis prior to phage-release could be too narrow in the bacteria at this pH level. The relative phage copy number generally increased over time at all pH levels investigated. At pH 5.5, the relative phage copy number was increasing dramatically over time, suggesting that substantial prophage induction had occurred. The *sea *gene copy number, however, was decreasing over time at pH 5.5. This could be due to cell lysis occurring upon prophage induction at this pH. At pH 5.0 and 4.5, a big increase in relative *sea *gene copy number was observed between the two last sampling points. This suggests that the prophage has been induced and the replicative form of the phage DNA is produced. However, at these low pH values, no great increase in SEA or phage copies were observed, suggesting protein synthesis was impaired.

In addition, the reason why the *sea *expression of *S. aureus *Mu50 at pH 5.5 was not as high as at pH 6.0, despite the fact that an even higher level of acetic acid was added, could also be because the acetic acid concentration here was on the limit of that tolerated by the organism. The growth rate of the culture at pH 5.5 was almost half of that at pH 6.0. The expression pattern at pH 5.5 was different from the patterns at the higher pH levels studied, in that it lacked the sharp expression peak in the transitional phase. At pH levels below 6.0, low amounts of SEA were produced. This supports the theory that pH 5.5 is close to the limiting pH of the bacterium. The SEA levels remained constant at pH 5.0 and pH 4.5 during the cultivation of Mu50, with a final SEA concentration of 62 ng/ml for both pH levels, indicating that no SEA production occured ≤ pH 5.0. This observation is supported by Barber and Deibel [32]. Using hydrochloric acid, they found that the lowest pH values that supported SEA biosynthesis in buffered BHI medium incubated aerobically was 4.9. SFP can be caused by as little as 20-100 ng of enterotoxin [33]. Levels higher than 100 ng/ml were detected at pH levels 7.0-5.5 in the mid-exponential growth phase.

## Conclusions

This study has shown that the food preservative acetic acid increases *sea *gene expression in *S. aureus*. At pH 6.0 and 5.5, maximal *sea *expression was observed. At pH 6.0 there was a marked shift in growth rate and phage production peaked at pH 5.5. These findings suggest prophage induction. At pH 5.0 and 4.5, the *sea *gene copy numbers increased dramatically during late stages of cultivation, but SEA levels and phage copy numbers were low indicating that protein synthesis was affected. It is our hypothesis that the acetic acid lowers the intracellular pH of *S. aureus*, activating the temperate phage and, as a consequence, boosts the *sea *expression. Our results support the theory proposed by other research groups that prophages not only facilitate the dissemination of virulence genes, but also take part in the regulation of the expression of the genes.

## Methods

### Culture conditions

The *S. aureus *strains used in this study were Mu50 (LGC Promochem, London, UK), MW2 (donated by Dr. T. Baba, Juntendo University, Tokyo, Japan), Newman (donated by Dr. H. Ingmer, Copenhagen University, Copenhagen, Denmark), RN4220 (Culture Collection University of Göteborg, Göteborg, Sweden), RN450 (donated by Dr. J. R. Penadés, Instituto Valenciano de Investigaciones Agrarias, Castellón, Spain), SA17 and SA45 (donated by the Swedish Institute for Food and Biotechnology, SIK, Göteborg, Sweden). All cultivations were performed in BHI (Difco Laboratories; BD Diagnostic Systems, Le Point de Claix, France) broth (with agitation) or agar at 37°C. *S. aureus *was transferred from glycerol stock to broth for overnight cultivation prior to the experiments. Broth (300 ml) was inoculated with a sufficient volume of *S. aureus *overnight culture to give an OD value at 620 nm (OD_620_) of 0.1 at the start of cultivation. Batch cultivations were then performed at different pH levels (pH 7.0, 6.5, 6.0, 5.5, 5.0, and 4.5) using in-house fermentors. The pH was set using acetic acid (Merck; Darmstadt, Germany), pH 7.0: 5.1 mM; pH 6.5: 12 mM; pH 6.0: 18 mM; pH 5.5: 28 mM; pH 5.0: 43 mM and pH 4.5: 93 mM final concentration of acetic acid, and maintained by adding sodium hydroxide (Merck) by automatic titration. The study was designed using several sampling points over time to visualize trends and all samples were analyzed three times. Where trend deviations were observed, cultivations were repeated to confirm the results.

The OD_620 _was measured to follow growth. All OD measurements were performed using a U-1800 spectrophotometer (Hitachi High Technologies Inc., Pleasanton, CA). Samples for quantitative reverse transcription polymerase chain reaction (qRT-PCR) analysis and enzyme-linked immunosorbent assay (ELISA) analysis, and intracellular-DNA and extracellular-DNA extractions were taken in the mid-exponential growth phase, in the transitional phase, i.e. between the exponential and stationary phases of growth, in the early stationary phase of growth, and in the late stationary phase of growth. At pH 5.0, samples were taken after 12, 27, 36 and 49 h of growth. At pH 4.5, samples were taken after 10, 24, and 30 h of growth.

Viable counts were determined in the late stationary growth phase to confirm OD_620 _measurements, except at pH 4.5, where viable counts were determined on each sampling occasion. Serial decimal dilutions of the bacterial cultures in physiological saline (Merck) solution were performed. The dilutions were plated on agar, incubated overnight and the CFU per ml was calculated.

### Primer and probe design

The forward primer, ESA-1, specific to *sea *was identified from the literature [34], and the reverse primer was designed in-house using LightCycler Probe Design^© ^software ver. 1.0 (Roche Diagnostics GmbH, Mannheim, Germany) (Table 2). Primers for the reference gene *rrn *were designed as the reverse primer of the *sea *gene. All primers were purchased from MWG Biotech AG (Ebersberg, Germany). Hybridization probes specific to *sea *and *rrn *were also designed using the LightCycler Probe Design^© ^software and purchased from TIB Molbiol GmbH (Berlin, Germany). The probes work in pairs. A donor probe labeled with fluorescein at the 3" end transmits the signal to an acceptor probe labeled with LCRed640/LCRed705 at the 5" end and the 3" hydroxy group is phosphorylated.

**Table 2:** Sequences and fluorescent dyes for primers and hybridization probes used for real-time PCR.

Target	Primer/probe	Nucleotide sequence (5' → 3')
*sea*	ESA-1	ACG ATC AAT TTT TAC AGC
	ToxA reverse	CCG AAG GTT CTG TAG AAG T
	ToxA-Fluo1	CCT TTG GAA ACG GTT AAA ACG AAT AAG AAA-FL^1^
	ToxA-Red1	LC-R640-TGT AAC TGT TCA GGA GTT GGA TCT TCA-p^2^
*rrn*	rRNA forward	TGT CGT GAG ATG TTG GG
	rRNA reverse	ACT AGC GAT TCC AGC TT
	Probe 1	GGA CAA TAC AAA GGG CAG CG-FL
	Probe 2	LC-R705-ACC GCG AGG TCA AGC A-p^3^

^1^The donor probe is labeled with fluorescein (FL) at the 3" end.

^2^The acceptor probe is labeled with LC Red640 (LC-R640) at the 5" end and the 3" hydroxy group is phosphorylated (p).

^3^The acceptor probe is labeled with LC Red705 (LC-R705) at the 5" end and the 3" hydroxy group is phosphorylated (p).

### *sea *expression analysis

Total RNA was extracted using phenol and chloroform as described by Lövenklev *et al*. [35], except that the RNA was re-suspended in 100 μl RNA storage solution (Applied Biosystems, Foster City, CA). First-strand cDNA was synthesized in two separate reverse-transcription assays using reverse primers specific to SEA and the reference gene 16S rRNA, as described previously [36], with 0.1 μg RNA in the reference gene assay and 0.5 μg RNA in the toxin gene assay.

Real-time PCR amplification was carried out on a LightCycler™ 1.0 instrument (Roche Diagnostics GmbH). The total volume of PCR mixture was 20 μl including 4 μl of template cDNA. The *sea *PCR mixture consisted of 1 × PCR buffer, 3.25 mM MgCl_2_, 0.2 mM each of dATP, dTTP, dCTP, and dGTP, 0.5 μM each of the forward and reverse primers, 0.05 U *Tth *DNA polymerase, and 0.3 μM of each hybridization probe. The *rrn *PCR mixture was the same as the *sea *PCR mixture, except that 0.15 μM of each hybridization probe was added. All reagents except the primers and probes were obtained from Roche Diagnostics GmbH. The water used was autoclaved ultrapure water. In order to detect the amplification of possible contaminants, a negative control consisting of water instead of DNA was added to the PCR. Genomic DNA was used as a positive control. The following PCR protocol was used: initial denaturation at 95°C for 1 min, followed by 45 cycles of denaturation at 95°C for 0 s (i.e., no hold at 95°C), primer annealing at 46°C (*sea*) or 48°C (*rrn*) for 5 s, and extension at 72°C for 25 s, with a single fluorescence measurement at the end of the extension step. The crossing point cycle for each transcript was determined using the second derivative maximum mathematical model in the LightCycler™ software (ver. 3.5) (Roche Diagnostics GmbH), and the amplification efficiency in the exponential phase was calculated using the equation of Klein *et al*. [37]. The *sea *gene assay was linear at 1.0 × 10^-6 ^to 6.3× 10^-8 ^g/ml RNA. The threshold cycle number of the reference gene varied <1.3 cycles in between samples. The efficiency was 0.96 ± 0.066 and 1.1 ± 0.075, respectively for the *sea *and the *rrn *assays.

The relative expression of the *sea *gene was calculated by relating the toxin gene expression to the constant expression of a reference gene, the 16S rRNA gene [38]. To determine the amplification efficiency and the log-linear range of amplification for each real-time PCR assay, the total RNA was serially diluted. The dilutions were reverse transcribed and amplified in the LightCycler™ instrument three times to obtain standard curves. Samples were also amplified three times. Equal amounts of total RNA from each sample were reverse transcribed to quantify the transcript levels of *sea*. The relative expression was calculated from the amplification efficiencies of each PCR assay and the crossing point deviation of the unknown sample versus a calibration sample, as described previously [38], see equation 1.(1)

### Phage induction analysis

Cell-DNA was extracted using a protocol described by Walsh *et al*. [39] modified to include a 20% Chelex^® ^(BioRad Laboratories; Hercules, USA) solution instead of 5%. 20 ng of DNA was added to the *sea *real-time PCR assay, see above.

Phage DNA was purified using zinc chloride as previously described by Santos [40] without previous DNase or RNase treatments. 200 ng of DNA was added to the *sea *real-time PCR assay, see above.

Induction of the bacteriophage using MC (Duchefa Biochemie, Haarlem, the Netherlands) was performed according to Resch *et al*. [41]. *S. aureus *overnight culture (0.2 ml) was added to 30 ml of fresh broth in 250 ml Erlenmeyer flasks. When cultures were in the mid-exponential phase of growth, MC was added to a final concentration of 0.5 μg/ml or 5 μg/ml, followed by continued incubation for 3 h. SEA concentrations, viable cell counts, and viable virus particles were determined. Cultures without addition of MC were used as controls.

The phage plaque assay was performed as described by France and Markham [42]. Supernatants from *S. aureus *cultures were spotted onto agar and the plates were then incubated at least overnight. *S. aureus *RN450 and RN4220 were used as receiver strains.

The relative *sea *gene copy number was calculated using equation 1. The relative phage copy number was calculated using the nominator part of equation 1.

### ELISA

A modified protocol was developed for ELISA analysis of SEA using affinity-purified sheep polyclonal antibodies based on Poli et al. [43]. A microtiter plate (Immulon^® ^2HB polystyrene, Flat Bottom Microtiter^® ^Plates, 96 wells solid; Thermo Electron Corporation; Waltham, MA) was coated with 100 μl/well of a solution containing 2 μg/ml SEA affinity-purified antibody (Toxin Technology, Inc.; Sarasota, FL) in coating buffer (0.1 M sodium carbonate, pH 9.6, Merck) and left at 37°C overnight. All sites were blocked with 185 μl blocking buffer (SuperBlock Blocking Buffer in PBS, pH 7.4, Pierce, Rockford, IL) for one hour at 37°C and at least one hour at 4°C. The plate was washed four times with washing buffer (0.05% Tween 20, BioRad Inc., in 10 mM PBS, Sigma-Aldrich, St Louis, MO). Standards or culture supernatants were loaded onto the plate (100 μl/well) at appropriate dilutions and incubated for 90 min at 37°C. As SEA standard, highly purified SEA staphylococcal enterotoxin from Toxin Technology Inc. (Sarasota, FL), was used. The plate was washed and the biotinylated antibody (Toxin Technology, Inc.), diluted 2000 × in assay buffer (50 mM PBS, 0.01% bovine serum albumin, Sigma-Aldrich, 0.1% Tween 20, 0.01% Thimerosal, Sigma-Aldrich, 1% milk powder, Semper, Sundbyberg, Sweden) was added (100 μl/well). The plate was incubated for one hour at 37°C and washed. NeutrAvidin™-linked alkaline phosphatase (ImmunoPure NeutrAvidin™, alkaline phosphatase conjugated, 0.9 mg/ml, Pierce; diluted 1000 × in assay buffer, no milk powder included) was added (100 μl/well), and the plate was incubated for 30 minutes at 37°C. The plate was washed and substrate (SIGMA*FAST*™ p-nitrophenyl phosphate tablets N2770, Sigma-Aldrich) was added (100 μl/well). The color was allowed to develop for 45 min in darkness and the optical density was determined using a microplate reader with a filter at 405 nm (Multiskan Ascent, Thermo Electron Corporation). Absorbance values (mean of triplicate wells) were plotted against toxin concentrations, and values were determined from linear regression. The detection limit was at 0.31 ng/ml of SEA.

### Nucleotide sequence analysis

The *sea *nucleotide sequences of six *S. aureus *strains (MRSA252 [GenBank: BX571856], MSSA476 [GenBank: BX571857], Mu3 [GenBank: AP009324], Mu50 [GenBank: BA000017], MW2 [GenBank: BA000033], and Newman [GenBank: AP009351]) were retrieved from GenBank (http://www.ncbi.nlm.nih.gov/Genbank/index.html, April 2009) and pairwise aligned using BioEdit v. 7.0.9.0 (Ibis Biosciences; Carlsbad, CA). DNA sequences (8 kb) upstream and downstream of the *sea *gene were also compared. The *sea *genes of all six strains have previously been annotated.

### Conventional PCR

Primers were designed to confirm the results of the nucleotide sequence analysis of *sea *and regions adjacent to the gene (Table 1). Two primer pairs were designed to distinguish between the two groups of nucleotide sequences, *sea*_1 _and *sea*_2_. Six primer pairs were designed to validate sequence differences found between strains in regions upstream and downstream of the *sea *gene. All primers were ordered from MWG Biotech AG. Genomic DNA from *S. aureus *Mu50, MW2, Newman, and SA45 was used as template. The total volume of PCR mixture was 50 μl including 200 ng template DNA. The PCR mixture consisted of 1 × PCR buffer, 2 mM MgCl_2_, 0.2 mM each of dATP, dTTP, dCTP, and dGTP, 0.2 μM each of forward and reverse primer and 2 U *Tth *DNA polymerase. All reagents except primers were obtained from Roche Diagnostics GmbH. The water used was autoclaved ultrapure water. In order to detect the amplification of possible contaminants, a negative control consisting of water instead of DNA was added to the PCR. The following PCR protocol was used: initial denaturation at 94°C for 4 min, followed by 30 cycles of denaturation at 94°C for 30 s, primer annealing at 47-55°C (see Table 1) for 30 s, and extension at 72°C for 1 min, with a final extension step at 72°C for 5 min. All amplifications were carried out using the Gene Amp 9700 thermal cycler (Perkin-Elmer Cetus; Norwalk, CT). The PCR products were visualized using 0.8% agarose (Bio-Rad Laboratories, Hercules, CA) gel electrophoresis according to Sambrook and Russell [44].

## Authors' contributions

NWC participated in designing the study, in carrying out the cultivations, the expression analysis and phage induction analysis, and in drafting the manuscript. RC participated in designing the study, and in carrying out the cultivations, the expression analysis, the phage induction analysis, the ELISA, and the nucleotide sequence analysis. DM participated in carrying out the cultivations, the expression analysis, phage induction analysis and the ELISA. AS participated in the phage induction analysis. JS and PR participated in designing the study and drafting the manuscript. All authors read and approved the manuscript.
